# *In vivo* production of a novel glycoconjugate vaccine against *Shigella flexneri* 2a in recombinant *Escherichia coli:* identification of stimulating factors for *in vivo* glycosylation

**DOI:** 10.1186/s12934-015-0195-7

**Published:** 2015-01-23

**Authors:** Michael M Kämpf, Martin Braun, Dominique Sirena, Julian Ihssen, Linda Thöny-Meyer, Qun Ren

**Affiliations:** Laboratory for Biointerfaces, Swiss Federal Laboratories for Materials Science and Technology (Empa), Lerchenfeldstrasse 5, CH-9014 St. Gallen, Switzerland; GlycoVaxyn AG, Grabenstrasse 3, 8952 Schlieren, Switzerland

**Keywords:** Glycoconjugate vaccine, *Shigella flexneri* 2a, Process optimization, High cell density culture, Recombinant *E. coli*

## Abstract

**Background:**

Glycoconjugated vaccines composed of polysaccharide antigens covalently linked to immunogenic carrier proteins have proved to belong to the most effective and safest vaccines for combating bacterial pathogens. The functional transfer of the N-glycosylation machinery from *Campylobacter jejuni* to the standard prokaryotic host *Escherichia coli* established a novel bioconjugation methodology termed bacterial glycoengineering.

**Results:**

In this study, we report on the production of a new recombinant glycoconjugate vaccine against *Shigella flexneri* 2a representing the major serotype for global outbreaks of shigellosis. We demonstrate that *S. flexneri* 2a O-polysaccharides can be transferred to a detoxified variant of *Pseudomonas aeruginosa* carrier protein exotoxin A (EPA) by the *C. jejuni* oligosaccharyltransferase PglB, resulting in glycosylated EPA-2a. Moreover, we optimized the *in vivo* production of this novel vaccine by identification and quantitative analysis of critical process parameters for glycoprotein synthesis. It was found that sequential induction of oligosaccharyltransferase PglB and carrier protein EPA increased the specific productivity of EPA-2a by a factor of 1.6. Furthermore, by the addition of 10 g/L of the monosaccharide N-acetylglucosamine during induction, glycoconjugate vaccine yield was boosted up to 3.1-fold. The optimum concentration of Mg^2+^ ions for N-glycan transfer was determined to be 10 mM. Finally, optimized parameters were transferred to high cell density cultures with a 46-fold increase of overall yield of glycoconjugate compared to the one in initial shake flask production.

**Conclusion:**

The present study is the first attempt to identify stimulating parameters for improved productivity of *S. flexneri* 2a bioconjugates. Optimization of glycosylation efficiency will ultimately foster the transfer of lab-scale expression to a cost-effective *in vivo* production process for a glycoconjugate vaccine against *S. flexneri* 2a in *E. coli*. This study is an important step towards this goal and provides a starting point for further optimization studies.

## Introduction

Gram-negative, non-motile, enteroinvasive *Shigella* bacteria are human pathogens that cause severe infection known as shigellosis. The disease is estimated to affect 165 million people annually, leading to approximately 1.1 million deaths per year (WHO). Especially children under the age of five living in environments with poor sanitation and hygiene conditions bear an elevated risk to contract an infection [1,2]. Among the different *Shigella* serotypes *S. flexneri* 2a is the most widespread strain worldwide and responsible for most endemic outbreaks in developing countries [3].

Vaccination has been proven as a powerful strategy to combat infectious diseases like shigellosis. In the last years several different approaches have been developed to combat *S. flexneri* 2a, including vaccination with attenuated or heat-killed *S. flexneri* 2a strains [4,5], recombinant outer membrane proteins [6,7], subunit-based vaccines [8] and glycoconjugate vaccines [9]. Particularly, conjugated vaccines composed of O-polysaccharide units of the lipopolysaccharide (LPS) covalently linked to immunogenic carrier proteins have attracted remarkable attention due to their inherent ability to evoke a T-cell dependent, long-lasting, serotype specific protective immunity. In contrary polysaccharide-only vaccines are often poor immunogens and elicit only T-cell independent, short-lived and low-affinity antibody responses [10,11]. It has already been demonstrated that glycoconjugates comprising O-specific polysaccharides of *S. flexneri* 2a covalently bound to *Pseudomonas aeruginosa* exoprotein A (EPA) are safe, immunogenic and efficacious in clinical phase III studies [12]. However, broad applicability of glycoconjugated vaccines has been hindered by the complex production process which relies either on sophisticated chemical synthesis to obtain, activate and couple the oligosaccharide to the carrier protein [9] or on cultivation of the bacterial pathogen in large cultures to obtain the desired O-specific polysaccharides which constitutes a major health and safety issue. Furthermore, processing of the chemical conjugates is laborious and requires different purification steps accompanied by substantial loss of target material, resulting in a low efficiency and cost-effectiveness [13]. Moreover, chemical crosslinking is highly unspecific, leading to low robustness and reproducibility of the production and consequently to difficulties in quality control of the vaccine.

Basic research of bacterial N-glycosylation resulted in the seminal discovery of the functional transfer of the *Campylobacter jejuni* N-glycosylation machinery in the standard prokaryotic host *E. coli* [14]. Key enzyme of this recombinant technology is the *C. jejuni* oligosaccharyltransferase PglB. It exhibits relaxed substrate specificity towards glycans from different origins [15] and is able to link these polysaccharides covalently to target proteins (e.g. immunogenic carrier proteins) that contain specific N-glycosylation sites [16]. Thereby tailor-made glycoconjugate vaccine candidates can be produced in non-toxic, engineered *E. coli* and purified in a simplified process from the bacterial periplasm as demonstrated recently for several polysaccharides of pathogens [17-20]. Depending on the polysaccharide substrate, there is a need for improving the glycosylation efficiency. Often a high percentage of the target protein remains unglycosylated, i.e., the glycoconjugate represents a small portion of the totally produced recombinant protein. A few studies describe the optimization of glycosylation efficiency by manipulation of the cellular metabolism [21-23].

In order to produce a more cost-effective vaccine for vaccination campaigns in developing countries, glycoconjugate yields can be optimized with respect to specific and volumetric productivity. High cell density cultivation (HCDC) of recombinant *E. coli* is a major strategy for maximizing volumetric productivity of recombinant proteins [24,25]. High cell densities can be reached by fed-batch cultivation, thereby reducing culture volume, enhancing biomass production and product recovery and hence reducing costs significantly. So far only one report describing a fed-batch bioprocess for *in vivo* production of a glycoconjugate vaccine against *S. dysenteriae* O1 in *E. coli* has been published [18].

In this study we report on (i) the establishment of an *in vivo* production system for the expression of a glycoconjugate vaccine against *S. flexneri* 2a in *E. coli*, (ii) the identification of critical parameters and cultivation conditions influencing the *in vivo* glycosylation efficiency and finally (iii) the transfer of the identified conditions to high cell density cultivations under controlled conditions to increase overall glycoconjugate yield. By applying a fed-batch process with the identified and optimized parameters the glycoconjugate yield was increased 46-fold compared to the shake flask cultures under non-optimized conditions.

## Results

### *In vivo* glycosylation of EPA with *S. flexneri* 2a O-polysaccharides

**Figure 1:**
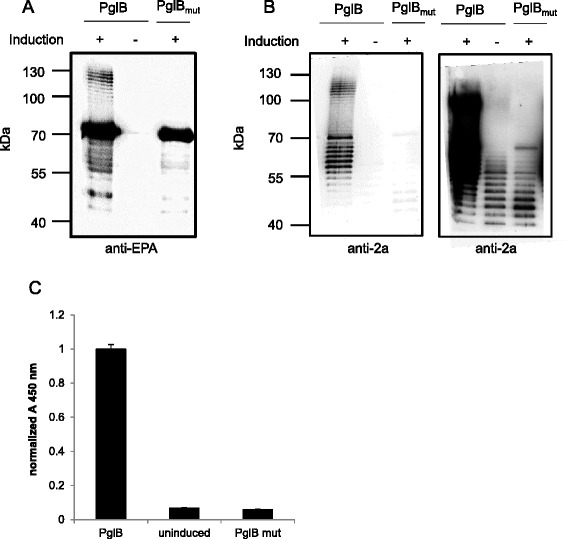
***In vivo*** **glycosylation of EPA with** ***Shigella flexneri*** **2a O-polysaccharides.** *E. coli* expressing *Shigella flexneri* 2a polysaccharides, EPA carrier protein and either PglB or PglB_mut_ were grown in shake flasks at 30°C and induced with 1 mM IPTG and 2 g/L arabinose or retained uninduced. 24 h post induction OD_600_-normalized periplasmic extracts were prepared and analyzed by Western blot with **A)** anti-EPA antibody or **B)** anti-2a antibody (**B**: right panel overexposed Western blot). **C)** Periplasmic extracts from **A)** and **B)** were analyzed and quantified by sandwich enzyme-linked immunosorbent assay (ELISA). Error bars represent the standard deviations of three biological replicates.

*In vivo* glycosylation of immunogenic carrier proteins with O-polysaccharides of bacterial pathogens in genetically engineered *E. coli* have been demonstrated to be a promising synthesis route for the production of glycoconjugate vaccines [17-20,26]. Due to the relaxed substrate specificity of the *C. jejuni* oligosaccharyltransferase PglB, various O-polysaccharides (O-PS) from Gram-negative and Gram-positive bacteria were successfully transferred to carrier proteins. We aimed at extending this palette of potential vaccines by producing a glycoconjugate against *Shigella flexneri* 2a, one of the most clinically relevant *Shigella* serotypes. In doing so, an *E. coli* strain was engineered harboring the O-antigen cluster of *S. flexneri* 2a integrated on its genome under control of its native, constitutive promoter and thereby replacing the endogenous *wb* cluster. Among other genetic modifications (see Materials and methods section) this strain lacks the O-antigen ligase *waaL*, thus preventing O-antigen transfer to the lipid A core, thereby promoting PglB-mediated transfer of the O-PS to the desired carrier protein. By co-expression of PglB under control of the IPTG-inducible P_*tac*_ promoter with a detoxified version of *Pseudomonas aeruginosa* exotoxin A (EPA) engineered with two N-glycosylation sites and an *E. coli* DsbA signal sequence for export to the periplasm, the *S. flexneri* 2a O-polysaccharides are supposed to be transferred to the respective sites of the carrier protein. As negative control, PglB was replaced by an inactive variant referred to as PglB_mut_ (W458A and D459A). After extraction of the periplasmatic fraction of induced *E. coli* cells, Western blot analysis either with anti-EPA antibody (Figure 1A) or with anti-*S. flexneri* 2a antibody (Figure 1B) was performed. A dominant band at 70 kDa was detected after hybridization with anti-EPA antibody in lane 1 and 3 (Figure 1A), representing the unglycosylated EPA carrier protein. A ladder of bands with higher molecular mass between 100–130 kDa was additionally and exclusively detected in lane 1 and constitutes glycosylated EPA protein with polysaccharide chains of different length generated by the coordinated action of the enzymes Wzy and Wzz. Wzy is responsible for polymerization of the O-PS and Wzz determines the extent of polymerization. In the uninduced sample in lane 2 only a very faint band at 70 kDa arising from leaky EPA expression from the P_*araBAD*_ promoter was visible. To elucidate whether also a specific antibody against *S. flexneri* 2a reacts with the proposed glycoprotein, Western blots of periplasmic extracts were hybridized with anti-2a antibody. The ladder of bands of high molecular mass was also detected with this specific antibody (lane 1, Figure 1B) while no glycoprotein was detected in the PglB_mut_ and uninduced samples. This confirmed the result from Figure 1A and showed that PglB is responsible and necessary for glycoprotein synthesis. Distinct bands below 70 kDa were also visible (Figure 1B, left and right overexposed panel, lanes 1, 2 and 3) and most likely represent undecaprenyl pyrophosphate (UPP)-linked O-antigens or degradation products. Although *Shigella flexneri* 2a O-PS were successfully transferred to EPA, most of the carrier protein remained unglycosylated (Figure 1A). Hence, a primary target of this study was to identify parameters and factors improving the yield of *S. flexneri* 2a glycoconjugates.

### Development of a reliable assay for quantification of glycoprotein

A prerequisite for optimization of glycoprotein yield is the availability of a reliable quantification method. In Figure 1A and 1B it was demonstrated that anti-EPA and anti-2a antibodies recognize EPA carrier and O-antigens, respectively. Therefore, we developed a sandwich enzyme-linked immunosorbent assay (ELISA) by coating high affinity 96-well plates with anti-EPA antibody as capture antibody and detecting bound glycoproteins from periplasmic fractions with anti-2a antibody (detection antibody). Appropriate dilutions of periplasmic extracts and antibody solutions were crucial to obtain high signal-to-noise ratios. Periplasmic samples from Figure 1A and Figure 1B were applied to the described ELISA format. The glycoprotein-containing sample resulted in a high readout at 450 nm while only negligible background signals were detected for the uninduced and PglB_mut_ samples, thus reflecting the Western blot results accurately (Figure 1C). With the described ELISA configuration the relative yield of EPA-2a in periplasmic extracts obtained from different cultivation conditions could be easily compared on the same ELISA plate. However, absolute quantification is challenging because unglycosylated EPA is competing with EPA-2a for capture antibody binding sites. Hence, purified EPA-2a does not represent an appropriate standard for this approach.

### Kinetics of EPA-2a production in shake flask and bioreactor

**Figure 2:**
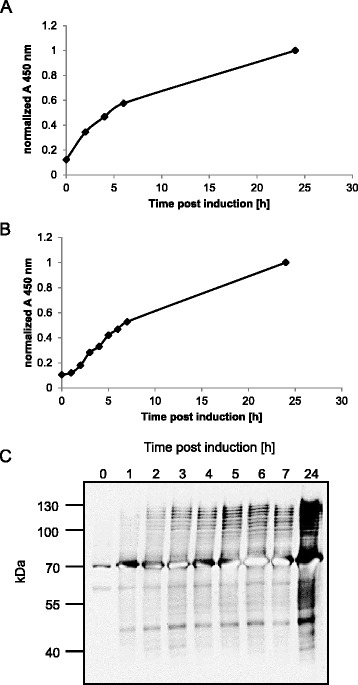
**Time course of** ***in vivo*** **glycosylation.** EPA-2a producing *E. coli* cells were cultivated at 30°C in shake flasks **(A)** or in a 1 L-bioreactor **(B and C)**. Samples were taken periodically after induction with 1 mM IPTG and 2 g/L arabinose followed by preparation of OD_600_-normalized periplasmic extracts and analysis by sandwich ELISA **(A and B)** or Western blot with anti-EPA antibody **(C)**.

After establishment of the quantification method for EPA-2a, the time course of *in vivo* glycosylation was monitored to obtain information about the optimal induction period at shake flask and bioreactor scale. Samples were taken periodically post induction and glycoprotein content was analyzed by ELISA after extraction. For both scales, specific productivity (i.e. glycoprotein content per cell) increased from 0 to 24 h with a maximum at 24 h (Figure 2A and 2B). Western blot analysis of bioreactor samples confirmed this result (Figure 2C). Since both profiles (Figure 2A and B) depict a similar progression of glycosylation per cell, the time-dependent production of EPA-2a glycoconjugates can be transferred directly from shake flask to bioreactor scale. In all following experiments engineered *E. coli* were induced for 24 h for EPA-2a production.

### Induction strategy affects product yield

**Figure 3:**
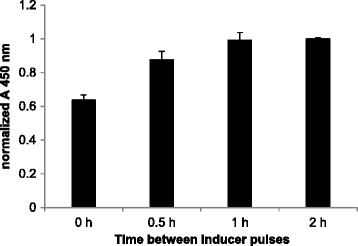
**Impact of order of induction on EPA-2a yield.** For EPA-2a production engineered *E. coli* were incubated at 30°C in shake flasks, and PglB and EPA carrier protein were induced either simultaneously (0 h) with 1 mM IPTG and 2 g/L arabinose, respectively, or EPA was induced at indicated time points after PglB induction. OD_600_-normalized periplasmic extracts were prepared 24 h post induction and analyzed by sandwich ELISA. Error bars represent the standard deviations of three biological replicates.

The oligosaccharyltransferase PglB is under control of the IPTG-inducible P_*tac*_ promoter while the carrier protein EPA is controlled by the P_*araBAD*_ promoter and its expression can be initiated by the addition of arabinose. This setup provides the opportunity to start expression of the two proteins at different time points. We therefore investigated whether the induction strategy has an impact on the specific productivity of EPA-2a by inducing PglB and EPA, either simultaneously or sequentially (first EPA, then PglB or *vice versa*). While inducing EPA 2 h before initiating PglB expression resulted in similar yield of glycoprotein compared to the simultaneously induced samples (Table 1), the sequential strategy with induction of PglB expression 2 h prior to that of EPA yielded a 1.6-fold increase of glycoconjugates (Figure 3 and Table 1). We further investigated different intervals from 0 to 2 h between both inducer pulses and found that glycoconjugate yield increased from simultaneous induction (0 h) to 0.5 h and reached a plateau at ≥1 h (Figure 3). The sequential induction did not lead to any significant change in the OD_600_ reached compared to simultaneous induction (data not shown). Thus, we conclude that a sequential induction strategy is advantageous for glycosylation efficiency and increases the specific yield of EPA-2a significantly (1.6-fold).

**Table 1:** **Induction strategies and influence on EPA-2a yield**

**Order of induction**	**Specific EPA-2a yield (A450 nm/OD**_**600**_ **)**
Simultaneous	0.63
Sequential (1. EPA 2. PglB)	0.55
Sequential (1. PglB 2. EPA)	1.0

EPA-2a expressing cells were incubated at 30°C. Cultures were induced either simultaneously with 1 mM IPTG and 2 g/L arabinose, or EPA was induced 2 h prior to PglB (1. EPA 2. PglB) or *vice versa* by the addition of 1 mM IPTG and 2 g/L arabinose. Values were obtained based on OD_600_-normalized periplasmic extracts.

### N-acetylglucosamine stimulates glycosylation efficiency

**Figure 4:**
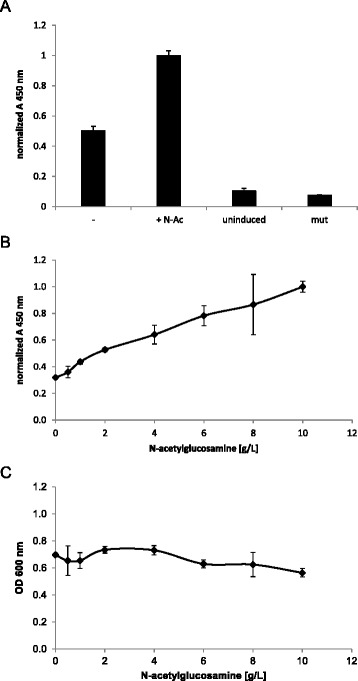
**Effect of N-acetylglucosamine on glycoconjugate synthesis. A)** Comparison of specific EPA-2a yield in the absence (−) or presence (+N-Ac) of N-acetylglucosamine (4 g/L) during simultaneous induction of PglB and EPA in shake flasks. **B)** EPA-2a expression in the presence of increasing amounts of N-acetylglucosamine (0 g/L – 10 g/L) in 96-deep well plates. Values were divided by the corresponding final OD_600_ before normalization to exclude that the beneficial effect of N-acetylglucosamine is only due to increase of biomass formation. *E. coli* cells were incubated at 30°C, and PglB and EPA were induced simultaneously for 24 h by addition of 1 mM IPTG and 2 g/L arabinose, respectively. **C)**. The final OD_600_ values in the presence of increasing amounts of N-acetylglucosamine (0 g/L – 10 g/L) in 96-deep well plates. Error bars represent the standard deviations of three biological replicates.

Serological classification of *Shigella* serotypes is based on the nature of the repeating unit (RU) of the O-specific polysaccharide moiety of the outer lipopolysaccharide layer (LPS), which acts as a major virulence factor for *Shigella* [27] and is the main target of the host adaptive immunity. The repeating unit of *S. flexneri* 2a is composed of a D-N-acetylglucosamine (D-GlcNAc) at the reducing end and three consecutive L-rhamnose (L-Rha) residues. This specific polysaccharide sequence motivated us to examine whether supplementation of the culture broth with monosaccharides occurring on the polysaccharide (D-GlcNAc, L-rhamnose) can stimulate glycoprotein synthesis. While addition of L-rhamnose exhibited only a marginal effect on the yield of EPA-2a (data not shown), D-GlcNAc increased the specific yield of glycosylated protein considerably. Analysis of OD_600_-normalized periplasmic extracts revealed that the amount of glycoconjugate per cell was improved 2-fold by the addition of 4 g/L D-GlcNAc compared to the sample without D-GlcNAc addition (Figure 4A). Since overexpression of recombinant proteins and particularly membrane proteins like PglB are considered to cause high stress to *E. coli* cells [28,29] and numerous publications pointed out that the disaccharide trehalose is synthesized as a stress-responsive factor [30-32], we investigated if supplementation of the culture medium with trehalose is also advantageous for EPA-2a synthesis. However, no significant effect on glycoprotein yield could be detected after trehalose addition (data not shown). To examine the specific effect of N-acetylglucosamine in more detail, a dose–response curve with different amounts of D-GlcNAc was recorded in 96-deep well plates. An increase of N-acetylglucosamine concentration led to improved specific productivity of EPA-2a up to 3.1-fold with 10 g/L GlcNAc (Figure 4B). This improved production of EPA-2a was not due to any effect of GlcNAc on the biomass as shown in Figure 4C, thereby confirming the specific, stimulating effect of N-acetylglucosamine for EPA-2a formation.

### Involvement of Mg^2+^ ions on glycoprotein synthesis

**Figure 5:**
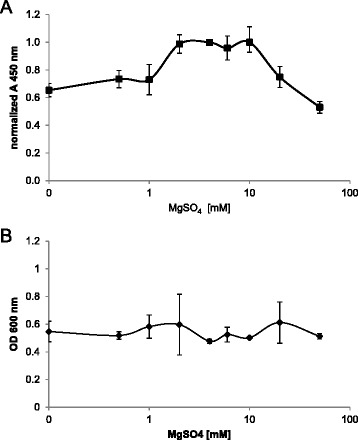
**Mg** ^**2+**^ **stimulates EPA-2a expression. A)** Culture medium was supplemented with different amounts of Mg^2+^, and EPA-2a was quantified in OD_600_-normalized periplasmic extracts by ELISA after incubation at 30°C and simultaneous induction with 1 mM IPTG and 2 g/L arabinose for 24 h in 96-deep well plates. **B)** Influence of different amounts of Mg^2+^ on the final OD_600_ values i in 96-deep well plates. Error bars represent the standard deviations of three biological replicates.

The transfer of O-PS to the desired target protein is mediated by the oligosaccharyltransferase PglB thereby forming an N-glycosidic linkage between the amide nitrogen of the acceptor asparagine and a distinct carbon of the monosaccharide at the reducing end of the polysaccharide chain. The precise reaction mechanism is not understood. However, it has been demonstrated that PglB and other oligosaccharyltransferases require divalent cations like Mn^2+^ or Mg^2+^ for activity [33,34]. Three acidic amino acids (D56, D154 and E149) in the catalytic pocket coordinate the binding of a total of three divalent cations. Mutations of these residues resulted in a decrease of glycosylation efficiency of > 50% [35]. Therefore, glycosylation efficiency of EPA with *S. flexneri* 2a O-PS was analyzed in the presence of different MgSO_4_ concentrations. An increase of Mg^2+^ ions in the range from 0 to 10 mM resulted in elevated glycoconjugate levels with an optimum of a MgSO_4_ concentration at 10 mM (Figure 5A). Mg^2+^ ions in the range from 0 to 10 mM did not lead to any significant change in biomass reflexed by OD_600_ values (Figure 5B). In the presence of higher MgSO_4_ concentrations (20 and 50 mM) a decrease in product yield was observed. These results demonstrate the promoting effect of Mg^2+^ on EPA-2a formation up to 10 mM.

### Synergistic effect of sequential induction and addition of N-acetylglucosamine

After identification of critical parameters e.g. the order of induction, the addition of N-acetylglucosamine and the presence of Mg^2+^ ions, the combined effect of optimized conditions was evaluated in shake flasks. Simultaneous induction of PglB and EPA carrier without addition of N-acetylglucosamine was used as the non-optimized conditions. Sequential induction of both proteins with a gap of 2 h between the inducer additions increased the yield 1.7-fold. The highest yield was obtained when the required proteins were induced sequentially and 10 g/L N-acetylglucosamine was added concomitantly to the culture broth, leading to a 2.6-fold increase of glycoconjugate yield compared to the non-optimized condition. The economical feasibility of the glycoconjugate production processes depends both on the attainment of high cell density and high levels of glycoprotein per cell. In order to produce glycoconjugate vaccines against *S. flexneri* 2a in sufficient amounts for immunization campaigns, the conditions optimized in this study were finally applied to high cell density cultures. EPA-2a producing cells were grown at 30°C until the optical density reached an OD_600_ value of 9. At this time 1 mM IPTG and 10 g/L N-acetylglucosamine were added to the culture. 2 h later the carrier protein EPA was induced by addition of 2 g/L arabinose, and feed medium was applied with a constant rate of 7.8 ml/h to provide the cells with required nutrients. Table 2 summarizes and compares directly the results from non-optimized and optimized shake flask experiments with bioreactor fermentations. While simultaneously induced shake flask cultures without N-acetylglucosamine reached a final OD_600_ of 1.9, sequential induction and the addition of 10 g/L N-acetylglucosamine led to an OD_600_ of 2.6 in shaken cultures and to an OD_600_ of 41.1 in fed-batch fermentations after 24 h of induction. The specific yield of optimized conditions determined by ELISA increased 2.6-fold in shake flasks and 2.1-fold in fed-batch cultivations, respectively. The obtained high cell density led to an increase of volumetric productivity by a factor of 46.2 compared to the non-optimized shake flask condition, while optimized conditions resulted in an increase of volumetric productivity by a factor of 3.6 in shake flask. The specific productivity was slightly decreased from a A_450_ value of 1.00 to 0.82 when optimized shake flask cultures were directly compared to high cell density fermentations, an effect which has also been observed previously [36].

**Table 2:** **Comparison of specific and volumetric EPA-2a yields in shake flask and high cell density cultivations**

**Type of cultivation**	**Induction strategy**	**N-acetylglucosamine g/L**	**OD**_**600**_ **at induction**	**Final OD**_**600**_	**Specific yield (A450 nm/OD**_**600**_ )*	**Volumetric yield (A450 nm/volume)**	**X-fold increase**
Shake flask	Simultaneous	-	0.8	1.9	0.38	0.72	-
Shake flask	Sequential	10	0.8	2.6	1.00	2.6	3.6
Bioreactor fed-batch	Sequential	10	9.1	41.1	0.82	33.7	46.2

*Values were obtained based on OD_600_-normalized periplasmic extracts.

## Discussion

Bacterial glycoengineering enables the *in vivo* glycosylation of immunogenic carrier proteins with bacterial O-polysaccharides, thus providing a novel platform for the production of tailor-made glycoproteins as safe and effective vaccines against various pathogens. Thereby the expensive and sophisticated chemical synthesis and coupling process is circumvented and cost-effective vaccines for immunization campaigns for developing countries can be realized. The most widely used enzyme technology exploits the relaxed substrate specificity of the *Campylobacter jejuni* oligosaccharyltransferase PglB towards diverse lipid-linked polysaccharides [15,37,38]. The PglB system was recently applied to produce glycoconjugate vaccines against *S. dysenteriae* serotype O1, *E. coli* O121, *Francisella tularensis* and *Brucella abortus* [17-20,39]. In this study we report the bioconjugation of *S. flexneri* 2a polysaccharides to the well-established immunogenic carrier exotoxoid A of *P. aeruginosa* (EPA). The *S. flexneri* 2a repeating unit consists of three rhamnose residues and a GlcNAc at the reducing end, similarly to other *Shigella* and *E. coli* serotypes, e.g. *S. dysenteriae* type 1 and *E. coli* O7 oligosaccharide [40,41]. We could show that *S. flexneri* 2a O-polysaccharides are a substrate for PglB-mediated transfer to EPA and that the resulting glycoproteins were recognized by a specific antibody targeting *S. flexneri* 2a glycans. After establishing the *S. flexneri* 2a system, our study aimed at identifying critical parameters stimulating glycoconjugate yield. Analysis of samples at different time points post induction showed that maximum glycoconjugate yield per cell was obtained after 24 h, even though high amounts of EPA carrier protein were already present in the periplasm 1 h after induction. A similar observation was made when EPA-*Shigella* O1 glycoconjugates were produced in *E. coli* [18]. This leads to the assumption that either PglB activity, O-polysaccharide assembly or polysaccharide precursor supply is rate-limiting. Induction time point and induction period plays a pivotal role in the expression of many recombinant proteins and the effectiveness of bioprocesses [42-44]. Upon addition of the inducer, overexpression of recombinant proteins consumes high amounts of essential biosynthetic precursors (e.g. amino acids, nucleotides). Thereby the cellular metabolism is negatively influenced which finally leads to multiple stress responses, reduced biomass formation and in turn to impaired recombinant protein expression [45-48]. Using the here described enzymatic protein glycan coupling technology, the situation is even more severe compared to the overexpression of single recombinant proteins. In this case, numerous components, i.e. oligosaccharyltransferase PglB, periplasmic protein carrier EPA and a whole set of enzymes required for lipid-linked O-polysaccharide synthesis have to be functionally expressed in a coordinate manner to enable maximum yield of the glycoconjugates. In the *S. flexneri* 2a system, the glycosyltransferase cluster was integrated in the host genome and constitutively expressed from its natural promoter. However, PglB oligosaccharyltransferase and EPA carrier protein were under control of the P_*tac*_ and P_*araBAD*_ promoters and were induced by IPTG and L-arabinose, respectively. The beneficial effect of sequential induction of PglB and EPA carrier expression observed in our study is probably related to the temporal separation of the overexpression of both components. This might relax the metabolic burden to some extent and allow a functional integration of PglB with its 13 transmembrane helices into the cell membrane by the precise interplay of translocases and insertases [49]. A correctly inserted, functional PglB, which is already present at induction of carrier protein expression, presumably is able to better transfer *Shigella flexneri* 2a glycans to EPA during export of the carrier polypeptide to the periplasm, possibly before folding. In the case of simultaneous or opposite induction, a larger portion of EPA remains unglycosylated due to the absence of functional PglB immediately after induction.

We have shown in this study that the addition of N-acetylglucosamine increased the yield of EPA-2a significantly. N-acetylglucosamine (GlcNAc) is an acetylated glucosamine derivative and plays a key role at the bacterial cell surface. Besides, it is also an important signaling molecule [50]. The first step in *Shigella flexneri* 2a O-polysaccharide assembly is the addition of one GlcNAc residue from the nucleotide activated sugar donor UDP-GlcNAc to the membrane-bound undecaprenyl pyrophosphate [51,52]. The UDP-GlcNAc is *de novo* synthesized in *E. coli* by the conversion of fructose-6-phosphate to glucosamine-6-phosphate [53], which is further processed by a glucosamine mutase to glucosamine-1-phosphate [54]. Acetylation of the latter intermediate leads to formation of GlcNAc-1-phosphate, which is the substrate for the final uridyl transfer generating UDP-GlcNAc [55]. It is assumed that synthesis of glucosamine-6-phosphate is the rate limiting step in UDP-GlcNAc synthesis [56]. In contrast to eukaryotic cells, bacteria are not able to synthesize GlcNAc-6-phosphate. However, when N-acetylglucosamine is present in the culture medium, the N-acetylglucosamine transporter NagE in the inner membrane of *E. coli* imports GlcNAc to the cytosol where it is immediately phosphorylated by the phosphotransferase system (PTS) to form GlcNAc-6-phosphate [57]. GlcNAc-6-phosphate is then converted *via* multiple enzymatic steps to GlcNAc-1-phosphate, which is finally activated by UTP to UDP-GlcNAc. Hence, the rate-limiting step of the *de novo* synthesis of glucosamine-6-phosphate is circumvented, which might result in an increased pool of the cytosolic activated sugar donor UDP-GlcNAc for glycan assembly and thus increase glycoprotein production. This hypothesis is supported by a recently published study, aiming at identifying genes beneficial for *in vivo* glycosylation of *C. jejuni* AcrA in a genome wide screen [21]. Among five other identified genes, the bi-functional enzyme Dxs, an UDP-N-acetylglucosamine pyrophosphorylase/glucosamine-1-phosphatase involved in UDP-GlcNAc precursor synthesis was identified and led to 1.6 fold increased AcrA glycosylation [21]. Interestingly, if GlcNAc was added to mammalian cells exogenously, it was also converted to UDP-GlcNAc, increasing the intracellular UDP-GlcNAc pool. The elevated level of UDP-GlcNAc is believed to result in enhanced N-glycan branching of glycosylated proteins in the Golgi apparatus [58].

In addition to the stimulating effect of N-acetylglucosamine, we found that Mg^2+^ ions are able to promote EPA-2a synthesis. Maximum EPA-2a yield was achieved by supplementation of the culture medium with 10 mM MgSO_4_. Divalent metal ions like Mg^2+^ and Mn^2+^ are essential co-factors for enzymatic reactions catalyzing the formation of phosphodiester bonds. The first reaction of the *S. flexneri* 2a O-polysaccharide assembly, the transfer of the GlcNAc moiety from UDP-GlcNAc to undecaprenyl pyrophosphate, is catalyzed by the integral membrane protein WecA in *E. coli. In vitro* assays with crude membrane extracts from overexpressing *wecA E. coli* cells demonstrated the absolute requirement of Mg^2+^ ions for the GlcNAc transfer [51]. Purified WecA from *T. maritima* revealed a Mg^2+^ -dependent activity profile with a maximum at 10 mM and inhibitory effects with higher Mg^2+^ concentrations similar to our data [59]. However, in contrast to the *in vitro* assay, EPA-2a was also produced in the absence of additional Mg^2+^ ions. This might be due to the complex medium components, yeast extract and soy peptone, in the culture medium which contain considerable amounts of metal ions. According to Liu et al. [60], the two glycosyltransferases WbgF and WbgG define the specific sequence of the repeating unit of the *S. flexneri* 2a O-polysaccharide by sequential addition of three rhamnose residues. The donor substrate for these glycosyltransferases is dTDP-rhamnose. Detailed biochemical characterization of WbgF and WbgG is lacking, so the requirement of divalent cations for their acitivity is speculative. The same holds true for GtrII, a glucosyltransferase attaching a glucose residue to rhamnose III, converting the repeating unit of *S. flexneri* serotype Y in 2a [61]. There is a general agreement that metal ions are required for oligosaccharyltransferase activity in different organisms [34,62,63]. A recent study on PglB from *Campylobacter lari* demonstrated the requirement of either Mn^2+^ or Mg^2+^ for DQNAT sequon binding of acceptor peptides [33]. It was shown that Mn^2+^ binds the acceptor with higher affinity than Mg^2+^, but this does not necessarily correlate with higher oligosaccharyltransferase catalytic activity. When essential amino acids (Asp-56 and Glu-319) involved in metal ion binding were mutated, glycosylation efficiency decreased dramatically and a double mutant was completely inactive [35]. Although Mn^2+^ is supposed to be the physiological cation for PglB, we performed our experiments in the presence of different MgSO_4_ which is an essential component of the production media for high cell density cultivations [64]. The increased EPA-2a yield in the presence of 10 mM MgSO_4_ might be generated by an overlapping effect of increased activity of involved glycosyltransferases (WecA, WbgF, WbgG) for O-polysaccharide assembly and enhanced catalytic activity of the oligosaccharyltransferase PglB. It would now be interesting to test whether the addition of extra Mn^2+^ also has a beneficial effect.

## Conclusion

The present study is the first attempt to identify stimulating parameters for improved productivity of *S. flexneri* 2a bioconjugates. Three major factors were identified and quantitatively analyzed. A sequential induction strategy with a 2 hour gap between both inducer pulses, the addition of 10 g/L N-acetylglucosamine and the presence of 10 mM MgSO_4_ were favorable for EPA-2a production. By applying these parameters to high cell density cultures, EPA-2a yield was increased 46-fold compared to initial shake flask conditions. It is likely that these factors are not *S. flexneri* 2a specific but enable increased productivities also of other glycoconjugates with similar structural features, i.e. a GlcNAc residue at the reducing end. However, this needs to be analyzed in further studies. Optimization of glycosylation efficiency will ultimately foster the transfer of lab-scale expression to a cost-effective and reasonable *in vivo* production process for a glycoconjugate vaccine against *S. flexneri* 2a in *E. coli*. This study is an important step towards this goal and provides a starting point for further optimization studies.

## Materials and methods

### Bacterial strains and plasmids

*E. coli* 1052 (W3110, F^−^, IN(rrnD-rrnE)1, *rph*^1^, Δ*wbbl,* Δ*wbbJ,* Δ*wbbK, gtrS::gtrII, ΔwaaL, wb* cluster::O-antigen cluster of *S. flexneri* 2457 T, *araBAD::cat)* (provided by GlycoVaxyn AG, Schlieren, Switzerland) harboring the O-antigen cluster of *S. flexneri* 2a under control of its native (constitutive) promoter on the genome was used as the production strain for all *in vivo* glycosylation experiments in this study. This strain also carries a genomic integration of the gene encoding glucosyltransferase GtrII, which attaches a glucose branch to the middle rhamnose residues essential for proper immune response, at the *gtrS* locus. The oligosaccharyltransferase PglB from *C. jejuni* was expressed from a spectinomycin-selectable, low-copy number expression plasmid (backbone pEXT21 [65], origin of replication IncW) under control of the IPTG-inducible hybrid promoter P_tac_. The PglB sequence was codon-optimized for expression in *E. coli* by gene synthesis (GenScript, Piscataway, NJ, USA). A glycosylation deficient PglB variant (PglB_mut_) harboring two point mutations (W458A and D459A) was used as negative control. For Western blot detection a hemagglutinin oligopeptide tag (HA) was genetically fused to the C-terminal end of the corresponding PglB sequences. As carrier protein, a detoxified version of *P. aeruginosa* exotoxin A (EPA, L552V, ΔE553) containing two engineered N-glycosylation sites (N262 and N398) was expressed from an ampicillin-resistant, high-copy number plasmid under control of the arabinose-inducible promoter P_araBAD_ (pEC415 [39]). For Sec-dependent secretion to the periplasm a DsbA signal peptide was genetically fused at the N-teminal end of EPA.

### Expression of EPA-2a in shake flasks

Small scale recombinant expression of the glycoconjugate vaccine EPA-2a was performed in 100 ml Erlenmeyer flasks (without baffles) filled with 50 ml medium. The complex medium used in this study was composed of 10 g/L yeast extract (Bacto yeast extract, BD, Le Pont de Claix, France), 20 g/L soy peptone (soy peptone A3 SC, Organotechnie, La Courneuve, France), 9 g/L KH_2_PO_4_, 5 g/L (NH_4_)_2_SO_4_, 1 g/L citric acid, 4 g/L glycerol, 10 mM MgSO_4_ and 10 ml/L trace element solution (10 g/L CaCo_3_, 20 g/L FeCl_3_*6 H_2_O, 1.5 g/L MnCl_2_*4 H_2_O, 0.3 g/L H_3_BO_3_, 0.25 g/L CoCl_2_*6 H_2_O, 0.15 g/L CuSO_4_, 0.5 g/L ZnCl_2_, 2 g/L NaMoO_4_, 84.4 g/L Na_4_EDTA*2 H_2_O, 20 ml/L, 37% HCl). To maintain plasmid stability the medium was supplemented with 100 μg/ml ampicillin and 80 μg/ml spectinomycin. Shake flasks were inoculated from overnight tube cultures to a starting OD_600_ value of 0.08 and incubated at 30°C and 160 rpm until cultures reached an OD_600_ of 0.6 – 0.8. PglB expression was induced by the addition of 1 mM IPTG, and the protein carrier EPA was induced by the addition of 2 g/L arabinose. For sequential induction, IPTG was added at an OD_600_ of 0.6 – 0.8 and 2 h later 2 g/L arabinose was added (if not stated otherwise). N-acetylglucosamine was always added concomitantly with IPTG. Induced bacteria cells were incubated over night before harvesting by centrifugation (6500 × g, 5 min, 4°C).

### Cultivation in 96-deep well plates

For screening of parameters that influence EPA-2a production, recombinant *E. coli* cells were grown in 96-deep well plates (DWPs) (VWR, order No. 732–0585) in 1.6 ml of the same medium used for shake flask cultures. DWPs were inoculated with an uninduced overnight shake flask culture to a starting OD_600_ of 0.05 – 0.1 and incubated at 30°C and 500 rpm in a specialized microplate incubator (Infors HT Microton, Bottmingen, Switzerland). When cultures reached an OD_600_ of 0.4 – 0.6, PglB and EPA expression was induced by adding 1 mM IPTG and 2 g/L arabinose respectively. N-acetylglucosamine was added simultaneously with IPTG (similar to shake flask experiments). After overnight incubation, 900 μl culture per well were transferred with a multi-channel pipette to a new DWP and harvested by centrifugation (1600 × g, 10 min, 4°C). Supernatant was withdrawn with a multi-channel pipette and periplasmic extracts were prepared as described below.

### Bioreactor fermentations

Optimization studies of high-cell density cultures were carried out in a 4-parallel bioreactor system (Infors HT, Multifors 2, Bottmingen, Switzerland) with a total vessel volume of 1 L. The composition of the complex medium was the same as described in the shake flask section, except that the initial carbon concentration was increased to 25 g/L glycerol. MgSO_4_, trace element solution and antibiotics were sterilized separately and added after autoclaving. The feeding solution consisted of 33 g/L yeast extract (Bacto yeast extract, BD, Le Pont de Claix, France), 67 g/L soy peptone (soy peptone A3 SC, Organotechnie, La Courneuve, France), 250 g/L glycerol, 10 mM MgSO_4_ and 10 ml/L trace element solution. The initial batch culture was started by inoculation of 0.5 L medium with an overnight seed culture to a final starting OD_600_ of 0.05. The pH was adjusted to 7.00+/−0.05 by the addition of 25% H_3_PO_4_ and 4 M KOH. Dissolved oxygen levels (DO_2_) were kept at 30% saturation by automated-enriching of the inlet air with pure oxygen. Bioreactors were stirred at 1000 rpm during the whole bioprocess. Foam formation was inhibited by the manual addition of the anti-foaming agent Antifoam 204 (Sigma-Aldrich, Buchs, Switzerland). PglB and EPA expression were induced by addition of 1 mM IPTG and 2 g/L arabinose, respectively. After induction, inducers were also added to the feed solution (concentration in feed: 1 mM IPTG, 2 g/L arabinose) to ensure their constant concentrations in the culture broth. Cell growth was monitored during the whole process by measuring the optical density (OD) at 600 nm using a UV-visible spectrophotometer (Genesys 6, ThermoSpectronic, Lausanne, Switzerland). Culture samples were diluted with deionized H_2_O until the final OD_600_ value was less than 0.4.

### Preparation of periplasmic extracts

In order to determine the specific productivity of glycoprotein production under altered conditions periplasmic proteins were isolated by an osmotic shock method [66]. In brief, cells corresponding to 2 (or 10) OD_600_ units were harvested by centrifugation at 6500 × g for 5 minutes and 4°C. Subsequently cell pellets were resuspended in 200 μl (or 1 ml) of chilled sucrose-lysozyme buffer (30 mM Tris–HCl pH 8, 20% w/v sucrose, 1 mM EDTA, 1 mg/ml lysozyme and complete protease inhibitor mix (Roche, Basel, Switzerland)) to a final OD_600_ of 10 and incubated on ice for 30 minutes. Periplasmic proteins were separated from cell debris and protoplasts by centrifugation at 6500 × g for 10 minutes at 4°C, and supernatant was withdrawn and stored at −20°C until further analysis.

### Enzyme-linked immunosorbent assay (ELISA)

For quantification of relative glycoconjugate yields in periplasmic extracts a sandwich ELISA was applied in a 96-well format (F96 MaxiSorp, Nunc). As capture antibody, protein G purified goat-anti-EPA antiserum (US Biological/Lucerna Chem AG, Lucerne, Switzerland) was diluted with 1 × PBS to a final concentration of 10 μg/ml, and microtiter plate wells were coated with 60 μl of capture antibody solution at 4°C overnight. All subsequent incubation steps were performed at room temperature. After four washing steps with 300 μl washing buffer PBST (1 × PBS, 0.05% Tween) separated by a 2 minutes incubation period under vigorous shaking using an automated microplate washer (Wellwash Versa, Thermo-Scientific, Zurich, Switzerland) wells were blocked for 2 h with 300 μl blocking solution (1 × PBS, 10% dry milk) followed by another washing procedure as described above. Subsequently, periplasmic extracts containing glycoproteins were diluted with dilution buffer (1 × PBS, 1% dry milk) to appropriate final dilutions of 1:100, 1:1000 or 1:10000, respectively, and 50 μl diluted periplasmic extracts were applied to ELISA plates and incubated for 1 h thereby allowing the antigen EPA to bind to the capture antibody. Unbound EPA-2a and unbound, unglycosylated EPA carrier protein were removed by four washing cycles with 300 μl washing buffer per well. Next 50 μl of a specific polyclonal antibody against the *S. flexneri* 2a polysaccharide chain developed in rabbit (rabbit-anti-2a; GVXN#92, GlycoVaxyn AG, Schlieren, Switzerland) were added as a 1:10000 dilution in 1 × PBS + 1% dry milk to the wells. After a 1 h incubation, plates were washed four times with washing buffer PBST to remove residual anti-2a antibody and probed for another hour with 50 μl of a 1:20000 dilution in 1 × PBS + 1% dry milk of the peroxidase-coupled detection antibody (goat anti-rabbit IgG-HRP, Bio-Rad, Reinach, Switzerland). Four final washing steps were performed before ELISA signals were developed with 100 μl Ultra-TMB-ELISA HRP substrate (Thermo-Scientific Pierce). The color reaction was stopped by addition of 100 μl of 1 N H_2_SO_4_ per well, and absorbance was measured in a 96-well photometer (BioTek, Synergy Mx, Lucerne, Switzerland) at 450 nm. The obtained A_450_ values allowed the relative comparison of EPA-2a in periplasmic extracts on the same plate. The highest A_450_ value was subsequently normalized to 1.

### Western blot analysis

Periplasmic extracts (5 μl) were supplemented by equal volumes of 2 × SDS-PAGE sample buffer (4% SDS, 20% glycerol, 10% 2-mercaptoethanol, 0.004% bromphenol blue, 0.125 M Tris HCl) and denatured at 95°C for 5 minutes. Samples were analyzed by 8% SDS-PAGE and transferred on a nitrocellulose membrane using the iBlot blotting system (Invitrogen, Carlsbad, USA). After blocking with 1 × PBS 10% milk for 1 h, membranes were probed with either rabbit anti-EPA antiserum (Sigma-Aldrich, Buchs, Switzerland) or rabbit anti-2a antiserum (GVXN#92, GlycoVaxyn AG, Schlieren, Switzerland), both applied as 1:20000 dilution in 1 × PBS 1% dry milk for 1 h. Prior to ECL-based chemiluminescent detection of EPA-2a glycoconjugates (ChemiDoc-It, UVP, Upland, USA) the membranes were hybridized with a peroxidase-coupled secondary antibody (goat anti-rabbit IgG-HRP, Bio-Rad, Reinach, Switzerland, 1:20000 in 1 × PBS 1% dry milk).
